# Correction: A combined crystallography and DFT study on ring-shaped cucurbit[*n*]urils: structures, surface character, and host–guest recognition

**DOI:** 10.1039/d3ra90125d

**Published:** 2024-01-16

**Authors:** Guoxun Zhu, Ao You, Huacan Song, Zhengquan Li

**Affiliations:** a Guangdong Provincial Key Laboratory of Chemical Measurement and Emergency Test Technology, Institute of Analysis, Guangdong Academy of Sciences (China National Analytical Center, Guangzhou) Guangzhou 510070 P. R. China 1539888623@qq.com lzq@fenxi.com.cn; b School of Eco-Environmental Technology, Guangdong Industry Polytechnic Guangzhou 510300 P. R. China 2015100029@gdip.edu.cn; c School of Chemical Engineering and Technology, Sun Yat-sen University Tangjia Zhuhai City 519082 P. R. China

## Abstract

Correction for ‘A combined crystallography and DFT study on ring-shaped Cucurbit[*n*]urils: structures, surface character, and host–guest recognition’ by Guoxun Zhu *et al.*, *RSC Adv.*, 2022, **12**, 10014–10019, https://doi.org/10.1039/D2RA00797E.

The author regrets that the funding information was incorrectly shown in the acknowledgements section of the original manuscript. The corrected funding acknowledgement is as shown below.

This work was supported by the following funding: Special Fund Projects of Guangdong Academy of Sciences for the Construction of Domestic First-class Research Institutions (2021GDASYL-20210103035), Education Commission of Guangdong Province (2020ZDZX2077), and Science and Technology Planning Project of Guangzhou (202102021119).

The Royal Society of Chemistry apologises for these errors and any consequent inconvenience to authors and readers.

## Supplementary Material

